# High-Density Lipoprotein Prevents Endoplasmic Reticulum Stress-Induced Downregulation of Liver LOX-1 Expression

**DOI:** 10.1371/journal.pone.0124285

**Published:** 2015-04-29

**Authors:** Dan Hong, Ling-Fang Li, Hai-Chao Gao, Xiang Wang, Chuan-Chang Li, Ying Luo, Yong-Ping Bai, Guo-Gang Zhang

**Affiliations:** 1 Department of Cardiovascular Medicine, Xiangya Hospital, Central South University, Changsha, Hunan, China; 2 Department of Cardiovascular Medicine, The Affiliated Hospital of Chengde Medical College, Hebei, China; 3 Department of Geriatric Medicine, Xiangya Hospital, Central South University, Changsha, Hunan, China; Duke University Medical Center, UNITED STATES

## Abstract

Lectin-like oxidized low-density lipoprotein receptor-1 (LOX-1) is a specific cell-surface receptor for oxidized-low-density lipoprotein (ox-LDL). The impact of high-density lipoprotein (HDL) on endoplasmic reticulum (ER) stress-mediated alteration of the LOX-1 level in hepatocytes remains unclear. We aimed to investigate the impact on LOX-1 expression by tunicamycin (TM)-induced ER stress and to determine the effect of HDL on TM-affected LOX-1 expression in hepatic L02 cells. Overexpression or silencing of related cellular genes was conducted in TM-treated cells. mRNA expression was evaluated using real-time polymerase chain reaction (PCR). Protein expression was analyzed by western blot and immunocytochemistry. Lipid uptake was examined by DiI-ox-LDL, followed by flow cytometric analysis. The results showed that TM induced the upregulation of ER chaperone GRP78, downregulation of LOX-1 expression, and lipid uptake. Knock down of IRE1 or XBP-1 effectively restored LOX-1 expression and improved lipid uptake in TM-treated cells. HDL treatment prevented the negative impact on LOX-1 expression and lipid uptake induced by TM. Additionally, 1–10 μg/mL HDL significantly reduced the GRP78, IRE1, and XBP-1 expression levels in TM-treated cells. Our findings reveal that HDL could prevent the TM-induced reduction of LOX-1 expression via inhibiting the IRE1/XBP-1 pathway, suggesting a new mechanism for beneficial roles of HDL in improving lipid metabolism.

## Introduction

Lectin-like oxidized low-density lipoprotein receptor-1 (LOX-1) is a specific cell-surface receptor for oxidized-low-density lipoprotein (ox-LDL) [1]. Increased LOX-1 activity in endothelial cells and macrophages may promote endothelial cell apoptosis and foam cell formation, leading to the progression of atherosclerosis (AS) [2]. LOX-1 is also located on the surface of liver cells [3.^4^], and overexpression of LOX-1 in mouse liver tissues has been reported to efficiently reduce the plasma ox-LDL level and significantly decrease the area of AS plaques [5], suggesting a promising protective function provided by hepatic expression of LOX-1. LOX-1 lowers the ox-LDL level in the circulation through hepatic uptake of ox-LDL, leading to reduced uptake of ox-LDL by endothelial cells and microphages in vascular walls and thereby reducing the risk of foam cell formation. These data highlight the possibility that effective removal of ox-LDL from circulation can be accomplished through competitive uptake in the liver.

Endoplasmic reticulum (ER) stress has been implicated in altering hepatic lipid metabolism [6] and may promote the progression of AS [7]. Ishiyama *et al*. have demonstrated that ER stress can upregulate LOX-1 expression in macrophages [8]. Furthermore, an elevated LOX-1 level has been observed in tumor necrosis factor-α (TNF-α)-regulated ER stress of endothelial cells [9]. Nonetheless, the impact of LOX-1 expression upon ER stress in liver cells is not well defined.

A low high-density lipoprotein (HDL) level is known to be a critical risk factor for cardiovascular diseases [10]. However, the association between HDL and cardiovascular disease risk is still being debated. A recent report indicates that a genetic variant that can induce a high plasma HDL cholesterol level does not reduce the risk of myocardial infarction [11]. Therefore, the impact of HDL against atherosclerosis needs to be further clarified.

In this study, we aimed to understand the exact effect of ER stress on LOX-1 expression and to determine the role of HDL on LOX-1 expression in a hepatic cell line under tunicamycin (TM)-induced ER stress [12]. We found that the LOX-1 expression and lipid uptake were decreased in TM-treated cells, which could be reversed by HDL. Our data indicate that HDL may reduce the negative impact of ER stress on LOX-1 expression through the IRE-1/XBP-1 pathway.

## Materials and Methods

### Reagents

RPMI 1640 medium, fetal bovine serum (FBS), penicillin, and streptomycin were obtained from Hyclone, Thermo Fisher Scientific Inc. (Salt Lake City, UT, USA). TM and anti-LOX-1 primary antibody were purchased from Sigma-Aldrich (St. Louis, MO, USA). HDL and ox-LDL labeled with the fluorescent probe 1,1′-dioctadecyl-3,3,3′,3′-tetramethyl-indocarbocyanine perchlorate (DiI-ox-LDL) were acquired from Guangzhou Zhongshan University School of Public Health, Guangzhou, Guangdong, China. Anti-GRP78, anti-IRE1, and anti-XBP-1 primary antibodies were purchased from Abcam (Cambridge, UK). The RevertAid first strand cDNA synthesis kit was obtained from Fermentas, Thermo Fisher Scientific Inc. (Salt Lake City, UT, USA). Polymerase chain reaction (PCR) primers and the SYBR green real-time PCR master mix kit were purchased from TaKaRa Holding Inc. (Tokyo, Japan). Only molecular biology- or cell culture-grade reagents were used.

### Cell culture

The normal human hepatic cell line L02 was obtained from the Cell Bank of the Institute of Biochemistry and Cell Biology, Shanghai, China. Cells were seeded in RPMI 1640 medium supplemented with 10% FBS, 100 IU/mL penicillin, and 100 μg/mL streptomycin. Cell cultures were maintained at 37°C in a humidified incubator supplied with 5% CO_2_. L02 cells at logarithmic growth phase from the fourth to sixth passages were plated for experiments.

### Transfection

In order to knock down the expression of target genes, L02 cells were transfected with the transfection mix containing Lipofectamine RNAiMAX transfection reagent (Invitrogen) and small interference RNAs (siRNAs): 10 nM Stealth Select RNAi (Invitrogen) directed against IRE-1 (HSS140847 and HSS176615), XBP-1 (HSS111391 and HSS111392), protein kinase R-like ER kinase (PERK) (HSS190343 and HSS190344), or activating transcription factor 6 (ATF-6) (HSS117915 and HSS177036). Stealth RNAi Negative Control Duplex (Low GC) was used as a negative control. The culture medium was replaced with fresh 1640 medium supplemented with 10% FBS at 12 h after transfection. Next, the cells were cultured with medium containing TM (1 μg/mL) for an additional 24 h. The expression levels of silenced genes were evaluated by real-time PCR and western blot analysis.

To overexpress LOX-1 in L02 cells, cells were transfected with pCMV6-XL5-LOX-1 plasmid encoding the full-length human LOX-1 cDNA, which was purchased from Origene Technologies (Rockville, MD, USA). FuGENE 6 transfection reagent (Roche Diagnostics, Mannheim, Germany) was used for preparing the transfection mix as described previously [13]. Control cells were transfected with pCMV6-XL5 empty vector. Forty-eight hours after transfection, L02 cells were exposed to TM (1 μg/mL) for an additional 24 h.

### Western blot analysis

Cells were collected and lysed with RIPA lysis buffer [50 mM Tris (pH 7.4), 150 mM NaCl, 1% Triton X-100, 1% sodium deoxycholate, and 0.1% sodium dodecyl sulfate (SDS)] (Beyotime, Jiangsu, China) for 30 min at 4°C. The total protein concentration of the cell lysate was determined using the bicinchoninic acid (BCA) reagent (Beyotime, Jiangsu, China). Protein samples (50–100 μg each) were loaded and separated by 10% SDS-polyacrylamide gel electrophoresis (SDS-PAGE). After gel electrophoresis, proteins were transferred to a polyvinylidene fluoride membrane for immunoblot analysis. After blocking in 5% nonfat milk, the membranes were probed with primary antibodies for GRP78 (1:500), LOX1 (1:500), IRE-1 (1:1000), XBP-1 (1:500), or β-actin (1:5000) and subsequently labeled with horseradish peroxidase-conjugated secondary antibody. The bands were visualized by the enhanced chemiluminescence reagent (Millipore, Billerica, MA, USA), and data were analyzed with a gel documentation system (Gel Doc1000 and Multi-Analyst version 1.1, Bio-Rad). All results were representative of at least three independent experiments.

### Immunofluorescence

Cells grown on a 48-well plate were fixed with 4% paraformaldehyde for 30 min at room temperature. After incubation with anti-GRP78 (1:400), anti-LOX1 (1:100), or anti-IRE-1 (1:300) primary antibody, the cells were then labeled with Dylight Fluor secondary antibody (EarthOx, SF, CA, USA) diluted in phosphate-buffered saline. Nuclei were counterstained with 4′,6-diamidino-2-phenylindole (DAPI). The stained cells were examined under a fluorescence microscope (Olympus, Tokyo, Japan).

### Real-time reverse transcription PCR analysis

Total RNA was isolated from L02 cells by TRIZOL extraction, and 2 μg of RNA from each sample was reversely transcribed into cDNA using the RevertAid first strand cDNA synthesis kit. The cDNA (5 μL) was amplified using SYBR Green Real-time PCR Master Mix and primers. The primers used for amplification were as follows: LOX-1, 5′-ACGGTTCTCCTTTGATGC-3′ (forward) and 5′-CTCTTAGGTTTGCCTTCTTC-3′ (backward); GAPDH, 5′-CTGCACCACCAACTGCTTAG-3′ (forward) and 5′-AGGTCCACCACTGACACGTT-3′ (backward). Amplification was carried out starting with an initial step at 95°C for 45 s, followed by 40 cycles of amplification (95°C for 5 s and 60°C for 31 s) by using an ABI 7500 real-time PCR system (Applied Biosystems, Foster City, CA, USA). GAPDH was used as an internal control, and data were expressed as the ratio of LOX-1 mRNA to GAPDH mRNA. All results were representative of at least three independent experiments.

### Evaluation of the hepatic lipid uptake

The ox-LDL uptake was measured by flow cytometric analysis. The cells were treated with DiI-ox-LDL (50 μg/mL) for 24 h. The average fluorescence intensity of DiI-ox-LDL in L02 cells was analyzed on a BD FACS Calibur instrument (BD, Franklin Lakes, NJ, USA).

### Statistical analysis

Data were expressed as means ± standard error of the mean (SEM). Multiple comparisons were conducted using analysis of variance (ANOVA), followed by the least-significant difference (LSD) test. P < 0.05 was recognized as statistically significant.

## Results

### Impact of ER stress on LOX-1 expression and lipid uptake ability in TM-treated L02 cells

**Fig 1:**
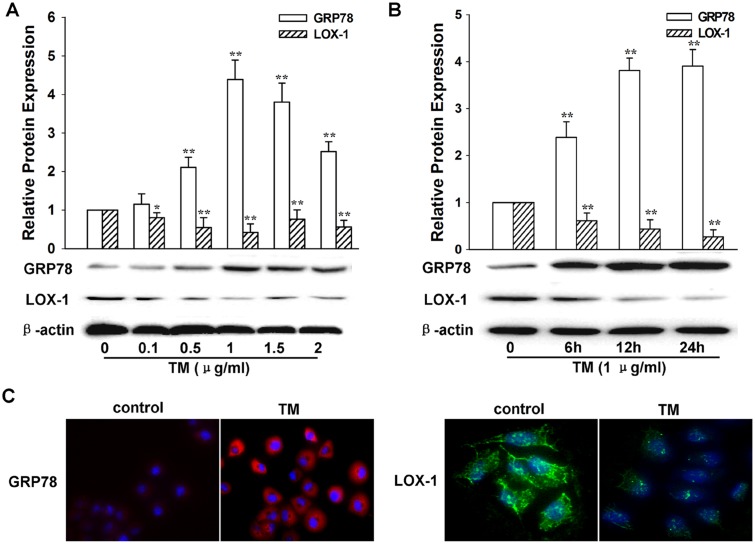
Impacts on GRP78 and LOX-1 expression by TM treatment in hepatic L02 cells. A: Expression levels of GRP78 and LOX-1 in hepatic L02 cells exposed to different concentrations (0.1, 0.5, 1, 1.5, or 2 μg/mL) of TM. Cells were harvested at 24 h after exposure to TM, and the expression levels of GRP78, LOX-1, and β-actin were examined by western blot. B: Impact of the time course of TM treatment on expression. The cells were exposed to TM (1 μg/mL) for 6, 12, or 24 h. Expression levels of GRP78, LOX-1, and β-actin were examined by western blot. C: Immunostaining of GRP78 (red) and LOX-1 (green) in L02 cells before (control) and after TM treatment (1 μg/mL, 24 h). Nuclei were counterstained with DAPI (blue). These data are representative of three independent experiments. *P < 0.05; **P < 0.01, compared with the control. TM: tunicamycin.

**Fig 2:**
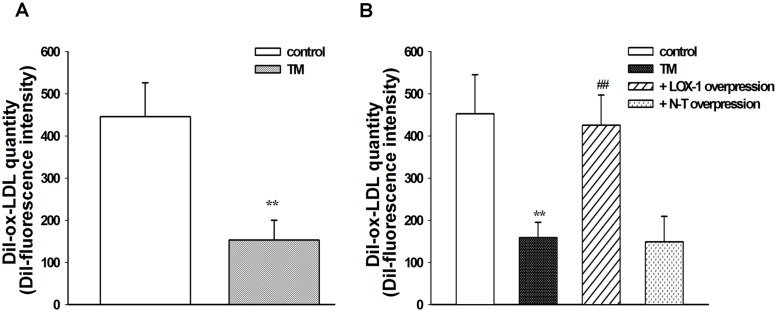
Impact of LOX-1 overexpression on lipid uptake capability in TM-treated hepatic L02 cells. A: Flow cytometric analysis of DiI-ox-LDL uptake capability in L02 cells before (control) or after 24 h TM treatment. Mean fluorescence intensity is presented. B: Cells were transfected with pCMV6-XL5-LOX-1 plasmid (LOX-1 overexpression) or pCMV6-XL5 empty vector (N-T mock transfection control) for 24 h before treatment with TM (1 μg/mL). Untransfected cells in the absence of TM were used as a control. These data are representative of three independent experiments. **P < 0.01 vs. control; ## P < 0.01 vs. TM treatment alone. TM: tunicamycin.

In order to understand the impact of ER stress on LOX-1 expression, L02 cells were treated with TM (0.1–2 μg/mL). Western blot analysis demonstrated that TM treatment dose-dependently upregulated the expression of ER chaperone GRP78 but downregulated LOX-1 expression at a dose of 0.1–1 μg/mL, whereas a high concentration of TM (1.5 or 2 μg/mL) seemed to have a unsaturated impact on inducing GRP78 expression or reducing LOX-1 expression (Fig 1A). Moreover, 1 μg/mL TM elevated the GRP78 level and decreased the LOX-1 level in a time-dependent manner within 24 h of incubation (Fig 1B). Immunocytochemical analysis confirmed that GRP78 upregulation and LOX-1 downregulation mediated by TM treatment peaked after incubation for 24 h at a dose of 1 μg/mL (Fig 1C). The DiI-ox-LDL uptake analysis (Fig 2A) showed that the lipid uptake activity of L02 cells was significantly reduced after TM exposure (*P* < 0.01, compared with the control). In addition, overexpression of LOX-1 in L02 cells by transfecting cells with pCMV6-XL5-LOX-1 plasmid reversed the TM-induced reduction of lipid uptake activity in cells (*P* < 0.01, compared with TM treatment alone), whereas no significant impact was found in cells transfected with empty control vector (*P* > 0.05, compared with TM treatment alone) (Fig 2B). These data suggest that ER stress triggered by TM may inhibit LOX-1 expression and decrease the lipid uptake ability of L02 cells. In addition, the capacity of liver cells to uptake ox-LDL could be enhanced by overexpression of LOX-1.

### Involvement of the IRE1/XBP-1 signaling pathway in TM-mediated downregulation of LOX-1 expression and lipid uptake function

**Fig 3:**
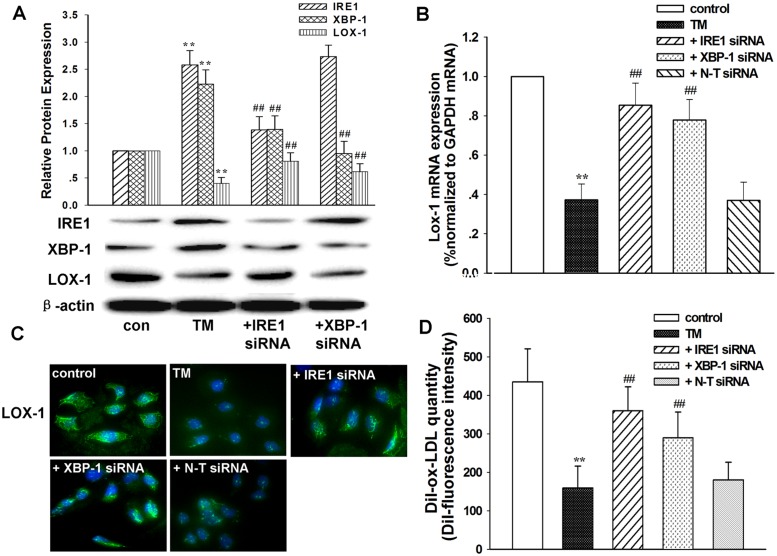
Involvement of the IRE1/XBP-1 signaling pathway in TM-mediated downregulation of LOX-1 and lipid uptake function. A: The effects of IRE1 and XBP-1 siRNA on TM-induced IRE1, XBP-1, and LOX-1 protein expression. Cells were transfected with siRNA targeting IRE1 or XBP-1. Protein expression levels were examined by western blotting. B–D: The effects of IRE1 and XBP-1 siRNA on the TM-induced LOX-1 altered expression. Cells were transfected with siRNA targeting IRE1, XBP-1, or negative control (N-T). The mRNA expression of LOX-1 was examined by real-time PCR (B). The LOX-1 protein expression (C) and lipid uptake (D) in L02 cells were examined by immunocytochemistry and DiI-ox-LDL uptake assays, respectively. Nuclei were counterstained with DAPI (blue). These data are representative of the results of three separate experiments. **P < 0.01 vs. control (0 μg/mL TM), ## P < 0.01 vs. TM (1 μg/mL). TM: tunicamycin.

In order to explore the underlying mechanism for TM-mediated downregulation of LOX-1, the expression levels of three major transducers of the unfolded protein response (UPR), namely IRE1, PERK, and ATF6, were determined. As shown in S1 Fig, cells with silenced IRE1 showed restored LOX-1 expression as compared with TM treatment alone (*P* < 0.01), while cells with the other two transducers (PERK and ATF6) silenced did not show restored LOX-1 expression. Hence, we next evaluated the involvement of the IRE1/XBP-1 pathway in this process. We found that TM treatment elevated the levels of both IRE-1 and XBP-1 (*P* < 0.01, compared with the control), and silencing of IRE-1 reduced the expression of XBP-1 in TM-treated L02 cells (Fig 3A). However, silencing of XBP-1 did not inhibit the TM-induced upregulation of IRE1. This evidence suggests that IRE-1 may serve as an upstream regulator of XBP-1. The knockdown of either IRE-1 or XBP-1 significantly restored the expression of LOX-1 in TM-treated cells (Fig 3A–3C). Additionally, knockdown of either IRE-1 or XBP-1 also reversed the reduced lipid uptake activity in TM-treated L02 cells (*P* < 0.01, compared with the TM treatment alone) (Fig 3D). No statistical difference was found in the expression levels of these genes after the cells were transfected with negative control siRNA (N-T siRNA). These data suggest that the IRE1/XBP-1 signaling pathway is involved in TM-mediated downregulation of LOX-1 expression and lipid uptake function in L02 cells.

### Effects of HDL on TM-mediated downregulation of LOX-1 and lipid uptake function

**Fig 4:**
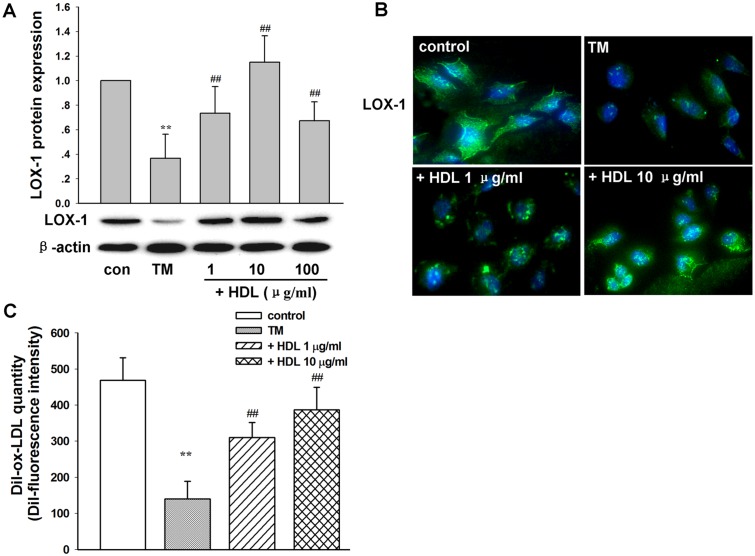
The effect of LOX-1 expression and lipid uptake induced by HDL addition to TM-treated hepatic L02 cells. A: Hepatic L02 cells were exposed to different concentrations (1, 10, and 100 μg/mL) of HDL together with TM (1 μg/mL) for 24 h. Cells without drug treatment were used as a control. Expression levels of LOX-1 and β-actin under different treatments were examined by western blot. B: Immunostaining of LOX-1 (green) in cells treated with TM (1 μg/mL), TM (1 μg/mL) plus HDL (1 μg/mL), or TM (1 μg/mL) plus HDL (10 μg/mL) for 24 h. Cells without drug treatment were used as a control. Nuclei were counterstained with DAPI (blue). C: Flow cytometric analysis of DiI-ox-LDL uptake capability. Mean florescence intensity is presented. These data are representative of three independent experiments. **P < 0.01 vs. control; ^##^ *P* < 0.01 vs. TM treatment alone. TM: tunicamycin.

As HDL plays an essential role in anti-atherosclerosis, we investigated the potential effect of HDL on TM-mediated downregulation of LOX-1 and lipid uptake function. Western blot analysis demonstrated that HDL incubation (1–100 μg/mL) prevented the inhibition of LOX-1 expression in TM-treated L02 cells (*P* < 0.01, compared with the TM treatment alone) (Fig 4A). The strongest protection of LOX-1 expression was detected in the presence of 10 μg/mL HDL in TM-treated cells. Immunocytochemical staining further confirmed that the restored LOX-1 expression peaked when 10 μg/mL HDL was used (Fig 4B). Moreover, the protected LOX-1 expression by HDL addition increased lipid uptake activity, which was detected in TM-treated cells by DiI-ox-LDL uptake analysis (*P* < 0.01, compared with the TM treatment alone) (Fig 4C)

### Influence of HDL on the IRE1/XBP-1 signaling pathway

**Fig 5:**
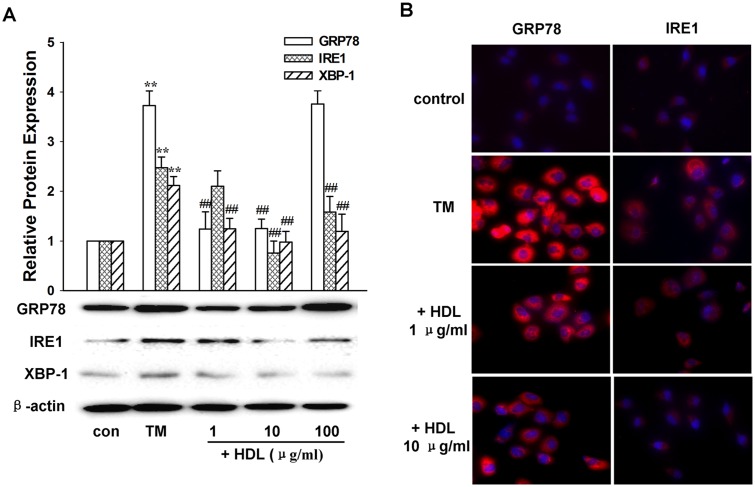
The impact of HDL on GRP78, IRE1, and XBP-1 expression levels in TM-treated cells. L02 cells were exposed to different concentrations (1, 10, and 100 μg/mL) of HDL together with TM (1 μg/mL) for 24 h. Cells treated with TM (1 μg/mL) alone were also included. Cells without drug treatment were used as a control. A: Expression levels of GRP78, IRE1, XBP-1, and β-actin were examined by western blot. B: Immunostaining of GRP78 (red) or IRE1 (red) in cells treated with TM (1 μg/mL), TM (1 μg/mL) plus HDL (1 μg/mL), or TM (1 μg/mL) plus HDL (10 μg/mL) for 24 h. Cells without drug treatment were used as a control. Nuclei were counterstained with DAPI (blue). Data are representative of three independent experiments. **P < 0.01 vs. control; ^##^ *P* < 0.01 vs. TM treatment alone. TM: tunicamycin.

Since the IRE1/XBP-1 signaling pathway regulates the TM-mediated downregulation of LOX-1 expression and HDL also regulates LOX-1 expression, we wondered whether HDL could impact the IRE1/XBP-1 signaling pathway; therefore, we analyzed the impact of HDL on the IRE1/XBP-1 expression level. As shown in Fig 5A, HDL (1–10 μg/mL) greatly reduced the GRP78, IRE1, and XBP-1 expression levels in TM-treated cells (*P* < 0.01, compared with the TM treatment alone); whereas no significant difference was found in the expression levels of the above proteins when the cells were exposed to 100 μg/mL HDL (*P* > 0.05, compared with the TM treatment alone). Similar results were obtained by immunocytochemical analysis (Fig 5B). These data suggest that the protection of LOX-1 expression and lipid uptake activity by HDL addition to TM-treated cells could be mediated through the IRE1/XBP-1 signaling pathway.

## Discussion

In our present study, we found that 1) TM treatment of hepatic L02 cells downregulated the LOX-1 expression as well as lipid uptake ability and such downregulations are associated with activation of the IRE1/XBP-1 signaling pathway; 2) HDL protected the LOX-1 expression level and hepatic lipid uptake efficiency in TM-treated cells; and 3) HDL may function via suppressing the IRE1/XBP-1 signaling pathway.

Recent studies have shed light on the role of ER stress in the pathogenesis and progression of AS [14]. Reducing ER stress through a macrophage lipid chaperone has been reported to alleviate AS [15]. In this study, TM-stimulated ER stress led to the increased expression of ER chaperone GRP78. LOX-1 is one of the most important receptors in the family of scavenger receptors and can be upregulated by a variety of stimuli, including ox-LDL, angiotensin II, proinflammatory cytokines, and sheer stress [16]. Therefore, we investigated the expression levels of LOX-1 in ER-stressed cells. LOX-1 is mainly expressed in endothelial cells and microphages residing in the vascular wall [17], and it is also expressed in human liver sinusoidal endothelial cells and possibly Kupffer cells [18]. Murine hepatocytes do support overexpression of LOX-1 mediated by infecting adenoviruses that carry the LOX-1 gene [5]. In this study, we detected LOX-1 expression in L02 cells by immunoblotting. We further found that TM significantly reduced the level of LOX-1 expressed in L02 cells. Overexpression of LOX-1 reversed the decreased lipid uptake function in the TM-treated cells, suggesting positive correlations between changing LOX-1 levels and lipid uptake efficiencies: the higher the LOX-1 expression level, the more efficient the lipid uptake function in hepatocytes. Our previous study showed that ox-LDL mediated ER stress and promoted endothelial cell apoptosis and AS via regulating LOX-1 expression [19]. In this study, we demonstrated that TM-induced ER stress significantly reduced hepatic LOX-1 expression, and decreased ox-LDL uptake and degradation. Collectively, severe ER stress may promote AS by increasing endothelial cell apoptosis and inhibiting lipid uptake by hepatocytes (S2 Fig).

**Fig 6:**
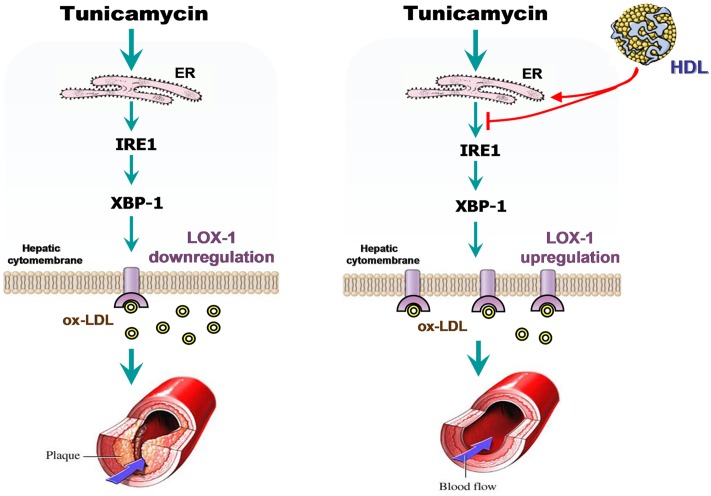
Proposed mechanism of HDL-mediated prevention of TM-induced downregulation of LOX-1 and lipid uptake function in hepatic cells. The ER stress inducer TM upregulates the expression of GRP78, thereby activating the IRE1/XBP-1 pathway. Activation of the IRE1/XBP-1 pathway leads to the downregulation of LOX-1 and lipid uptake function in hepatic L02 cells. HDL upregulates the TM-induced decrease of LOX-1 expression and hepatic lipid uptake, which is possibly mediated via inhibiting the IRE1/XBP-1 pathway.

ER stress induced by the accumulation of misfolded proteins in the ER initiates the UPR [20]. We found that only IRE1 is capable of reversing the LOX-1 level in TM-treated cells among the three major transducers of the UPR (IRE1, PERK, and ATF6) [7]. This finding indicates that only the transducer IRE1 participates in TM-mediated LOX-1 downregulation. Similar to the impact of IRE1, silencing of XBP-1, the downstream effector of IRE1, also reversed the lowered expression of LOX-1. In addition, knockdown of either IRE1 or XBP-1 improved the lipid uptake activity in TM-treated cells. These data suggest participation of the IRE1/XBP-1 pathway in regulating LOX-1 expression and lipid uptake function in TM-treated cells (Fig 6). Thus, inhibition of the IRE1/XBP-1 pathway may improve lipid metabolism by upregulating LOX-1 expression. Consequently, activation of the IRE1/XBP-1 pathway may downregulate LOX-1 expression.

It is well accepted that HDL protects against AS by removing cholesterol from peripheral tissues and blood vessels, inhibiting the oxidation of LDLs, limiting the generation of foam cells, and preventing inflammation, apoptosis, and vascular endothelial damage. Epidemiological and clinical studies indicate that a low level of HDL-cholesterol might be linked to an increased risk of cardiovascular diseases [21]. Consistent with this evidence, we found that HDL at a dose of 10 μg/mL was the most effective treatment to protect the lipid uptake activity in TM-treated L02 cells. The HDL-mediated cytoprotection against ER stress has been widely studied in various cell types. For instance, HDL is reported to protect pancreatic β-cells against ER stress by inhibiting the XBP-1/C/EBP-homologous protein (CHOP) signaling pathway and restoring protein folding and trafficking [22]. In human endothelial cells, HDL prevents ox-LDL-induced activation of the ER stress sensors, including inositol-requiring kinase 1α, eukaryotic translation initiation factor 2α, and ATF-6, and subsequent activation of the proapoptotic regulator CHOP [23, 24]. Moreover, HDL inhibits ER stress and promotes lipid efflux from lipid-loaded macrophages [25]. To the best of our knowledge, this study is the first to reveal that low (1 μg/mL) and medium (10 μg/mL) doses of HDL prevent the TM-induced upregulation of GRP78, IRE1, and XBP-1, but protect against TM-induced LOX-1 downregulation. However, increasing the HDL concentration to 100 μg/mL appeared to have an unsaturated impact on restoring LOX-1 expression. These results were in accordance with failed clinical trials with HDL, which showed that simply increasing the plasma concentration of HDL was less effective than expected.

Therefore, we conclude that low and medium concentrations of HDL (1–10 μg/mL) are sufficient to inhibit the IRE1/XBP-1 signaling pathway and upregulate LOX-1, contributing to the improved lipid uptake in hepatic L02 cells (Fig 6). In addition to HDL-dependent reverse cholesterol transport, our data suggest a possible new anti-atherogenic mechanism provided by HDL, which can neutralize the negative impact on the LOX-1 expression by ER stress to mitigate AS risk resulting from an elevated ox-LDL level. Our data also suggest that effective removal of ox-LDL from circulation can be accomplished through competitive uptake in the liver, which expresses LOX-1. Increasing the LOX-1 expression in the liver may have the therapeutic potential to lower the risk for AS by reducing the plasma ox-LDL level; in addition, it could lead to the reduced formation of foam cells in the artery walls. Currently, it is unclear which component in HDL particles is likely required to protect cells from experiencing the ox-LDL uptake disorder triggered by ER stress. Future studies will focus on understanding the detailed roles and molecular mechanisms of HDL in AS progression using an animal model.

## Supporting Information

S1 Fig The effects of UPR sensor siRNA on TM-induced LOX-1 downregulation. Cells were transfected with siRNA targeting three UPR sensors: IRE1, PERK, and ATF6. Stealth RNAi Negative Control Duplex was used as a negative control (N-T siRNA). The expression of LOX-1 mRNA was examined by real-time PCR.
(TIF)

S2 Fig Severe ER stress promote AS by increasing endothelial cell apoptosis and inhibiting lipid uptake by hepatocytes. (TIF)
