# Supplementary figures and images for: High-Density Lipoprotein Prevents Endoplasmic Reticulum Stress-Induced Downregulation of Liver LOX-1 Expression
Source: PLoS One. 2015 Apr 29;10(4):e0124285. doi: 10.1371/journal.pone.0124285 (PMC4414515; doi:10.1371/journal.pone.0124285)

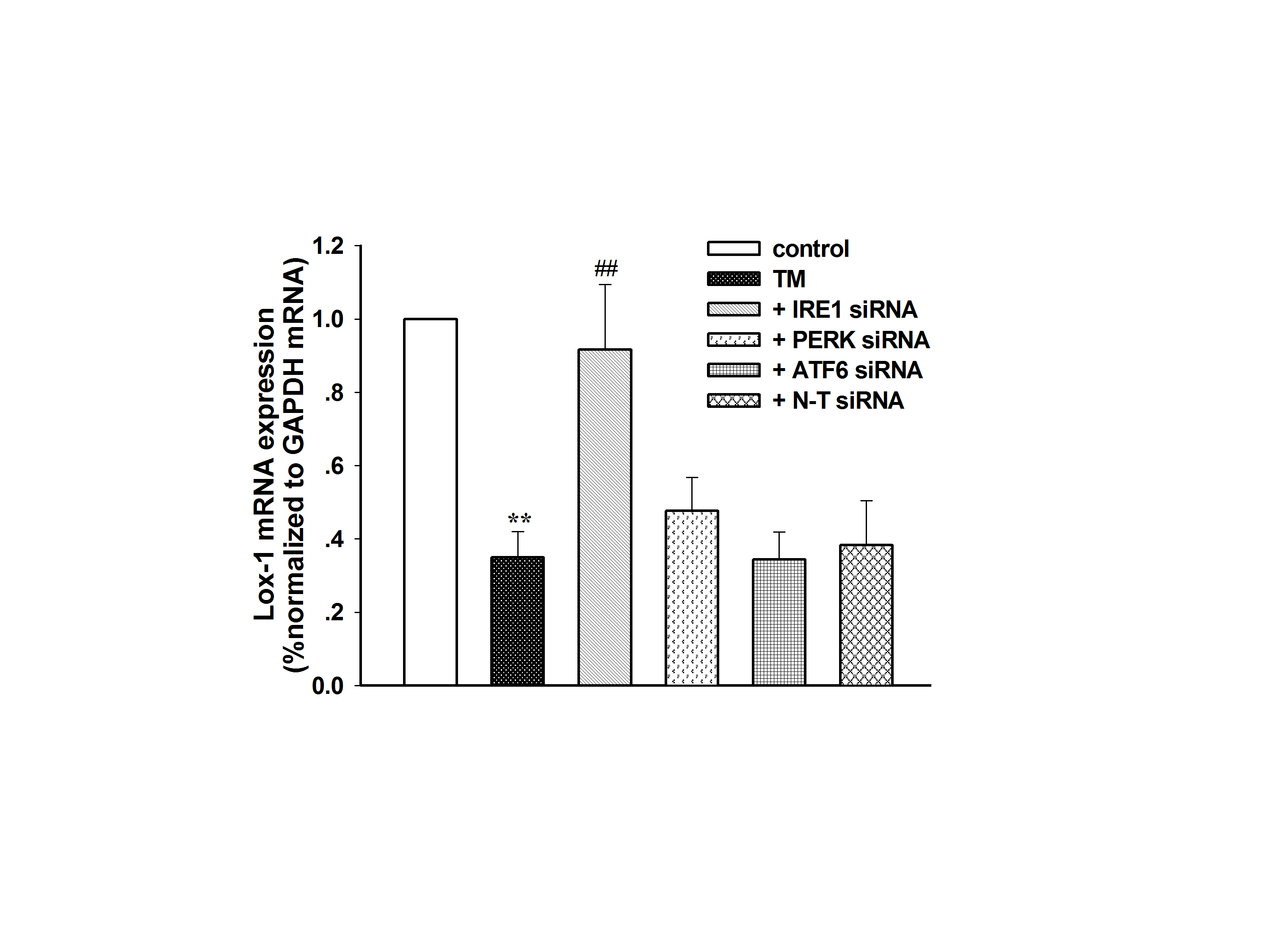

Supplement: S1 Fig — Cells were transfected with siRNA targeting three UPR sensors: IRE1, PERK, and ATF6. Stealth RNAi Negative Control Duplex was used as a negative control (N-T siRNA). The expression of LOX-1 mRNA was examined by real-time PCR. (TIF) [file pone.0124285.s001.tif]

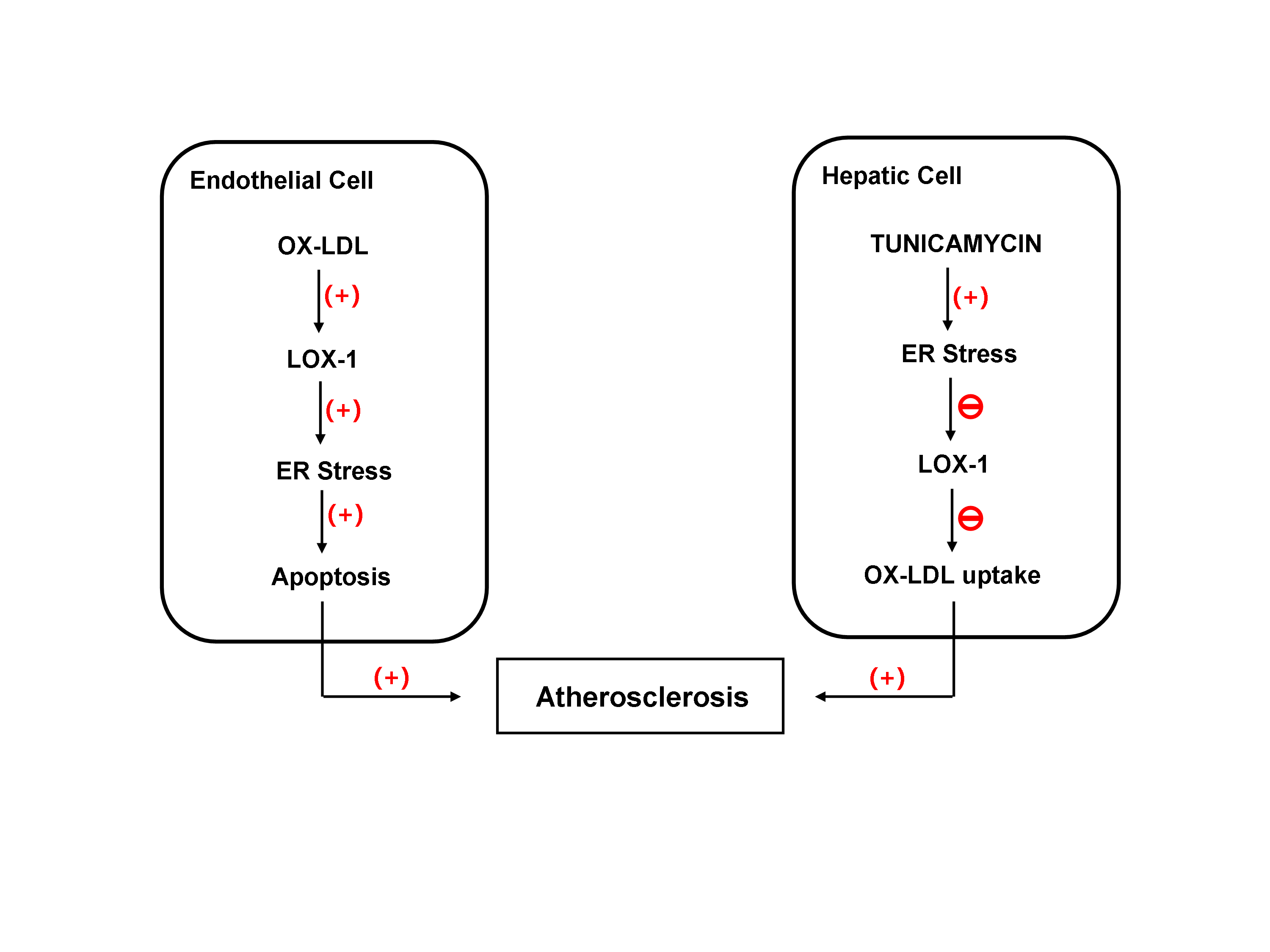

Supplement: S2 Fig — (TIF) [file pone.0124285.s002.tif]
